# Role of Probiotics in *Helicobacter pylori* Eradication: Lessons from a Study of *Lactobacillus reuteri* Strains DSM 17938 and ATCC PTA 6475 (Gastrus®) and a Proton-Pump Inhibitor

**DOI:** 10.1155/2019/3409820

**Published:** 2019-04-01

**Authors:** Maria Pina Dore, Stefano Bibbò, Giovanni Mario Pes, Ruggero Francavilla, David Y. Graham

**Affiliations:** ^1^Dipartimento di Medicina Clinica e Sperimentale, University of Sassari, Sassari, Italy; ^2^Department of Interdisciplinary Medicine, University Hospital of Bari, Bari, Italy; ^3^Department of Medicine, Michael E. DeBakey VA Medical Center and Baylor College of Medicine, Houston, TX, USA

## Abstract

**Background:**

Meta-analyses involving >4000 subjects with probiotics added to antimicrobial *Helicobacter pylori* eradication therapy have reported a mean increase in the eradication rate of 12 to 14%. It is unclear how to translate that result into clinical practice.

**Aim:**

To evaluate whether administration of *Lactobacillus reuteri* plus a PPI without antibiotics would eradicate *H. pylori* infections.

**Methods:**

This was a double-blind placebo-controlled randomized 2-site study of *L. reuteri* (Gastrus®) at a dose of 2 × 10^8^ CFU, 7 times per day, or matching placebo plus 20 mg pantoprazole b.i.d. for 4 weeks. Cure was defined by negative ^13^C-UBT, 4 weeks after therapy. Sample size required ≥50% cure rates for using probiotics as a clinically useful monotherapy.

**Results:**

Recruitment was halted after 56 subjects because of the low cure rate; there were 8 dropouts; 48 subjects completed therapy (71% women, average age 49 years). The cure rates per protocol were 3/24 (12.5%; 95% CI 2.6–32%) with *L. reuteri* vs. 1/24 (4.1%) with placebo. Side effects (most often diarrhea) occurred infrequently (in 5/28 vs. 3/28; active vs. placebo therapy) (*P*=0.53).

**Conclusion:**

*L. reuteri* plus a PPI therapy was unable to provide a clinically important rate of *H. pylori* eradication. The cure rate albeit low (12.5%) was essentially identical to that achieved when probiotics were added to antibiotic therapy. The incremental improvement was additive and independent of antimicrobial resistance or antibiotics use. Probiotics can reliably increase the cure rate to ≥90% only in regimens achieving cure rates of ∼80%. This trial is registered with NCT03404440.

## 1. Introduction

Probiotics are defined as live microorganisms which, when administered in adequate amounts, confer a health benefit on the host. Research in the use of probiotics as medical foods had a slow start and only achieved exponential growth after 2000 [1], and probiotics are now used in the treatment of a wide variety of conditions ranging from vaginitis to allergies and sepsis. Probably, the widest use has been in gastroenterology including using as an adjuvant for the treatment of *Helicobacter pylori* infections [2].

**Figure 1:**
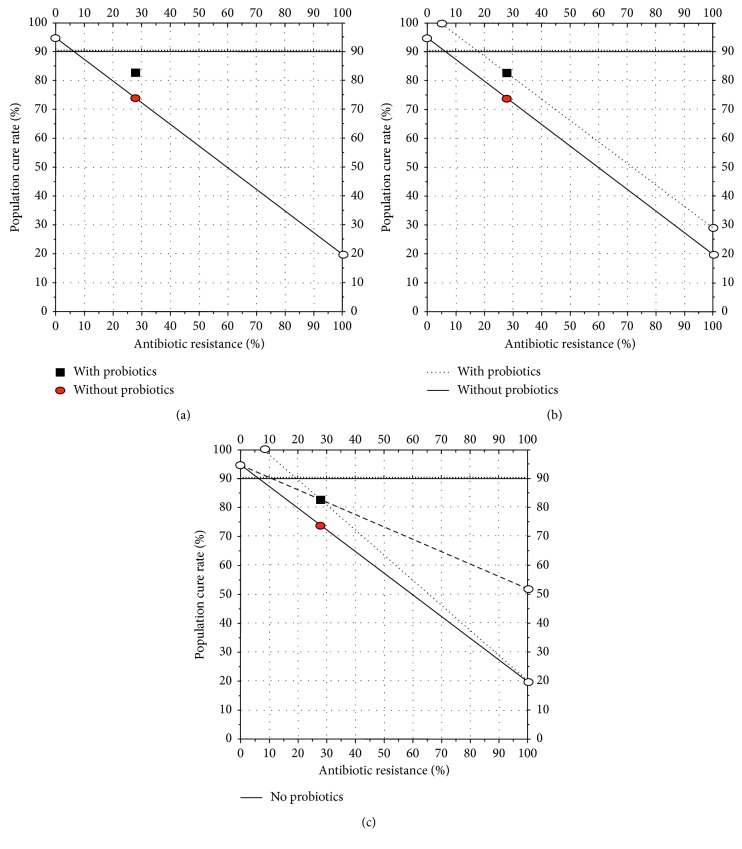
(a) Hp-nomogram [9] showing theoretical cure rates with 7-day triple therapy in the absence and presence of resistance plotted based on a cure rate of 95% with susceptible infections and 20% with resistant infections consistent results in western populations. Using the data from the meta-analysis [4], the overall cure rate PP was 74.1% (circle) which would be equivalent to a proportion with resistance of approximately 28%. The results with probiotics are plotted (square) as the same resistance rate as they came from the same population. (b) Hp-treatment nomogram showing the results plotted as an additive effect (i.e., the cure rate of the overall population plus 9.1% which is the absolute delta that achieved a 12.1% increase from 74.1 to 83.5% as shown in Figure 1(a) based on [4]). The formula would be ((cure rate with susceptible infections) × (proportion with susceptible infections) + (cure rate with resistant infections) times (proportion with resistant infections)) + (absolute percent increase with probiotic). (c) Hp-treatment nomogram showing the alternate ways the outcomes could be plotted if the increase with the probiotic had been entirely dependent on the increased effect among those with susceptible infections (dotted line) or among those with resistant infections (dashed line). The experiment described above showed that the best representation is the one shown in Figure 2.

One of the most common research questions has been to investigate whether the addition of probiotics to traditional triple *H. pylori* eradication therapy would increase the cure rate. The studies have typically involved populations which have increased antimicrobial resistance resulting in a reduced cure rate, which provided the ability to identify whether the addition of a probiotic results in improvement. The results of these studies have been summarized by meta-analyses, a number of which have been published in the last 2 years [2–8]. On average, the addition of a probiotic to antimicrobial therapy (most often a combination therapy consisting of a proton-pump inhibitor (PPI), clarithromycin, and amoxicillin) has resulted in an increase in the cure rate of 10% to 14%. The lack of corresponding susceptibility data has prevented detailed analyses of whether the increase is limited to a subpopulation (e.g., susceptible or resistant infections) or is caused by a similar increase in both (e.g., an additive effect). The outcome of *H. pylori* triple therapy can be estimated based on the formula ((cure rate of susceptible infections) × (proportion with susceptible infections) + (cure rate with resistant infections) × (proportion with resistant infections)), which is easily visualized using the Hp-treatment nomogram [9]. For example, a recent meta-analysis showed that probiotics improved treatment results by an average of 12.2% (1,786/2,140 [83.5%] vs. 1,602/2,162 [74.1%]) per protocol, resulting in an absolute difference of 9.1% [4].  Figure 1(a) shows these results plotted on an Hp nomogram based on a cure rate of 95% with susceptible infections and 20% with resistant infections were consistent with western results. It shows that the, in the trials cited, the proportion with resistance was approximately 28%.  Figure 1(b) shows the results plotted as an additive effect (of 9.1%) irrespective of the presence or absence of resistance as is seen with bismuth cotherapy.  Figure 1(c) shows the outcomes if the effect was entirely dependent on those with susceptible infections (dotted line) or those with resistant infections (dashed line). In both of those instances, the percent improvement would depend on the proportion with resistance. It is impossible to choose among these (or other choices such as the overall cure rate plus 12%) without knowing the outcome with either the susceptible and/or the resistant populations.

Importantly, the addition of the probiotic has rarely achieved an overall cure rate of ≥90%. Interpretation of the available meta-analyses is also complicated as they combined antimicrobial regimens that differed in terms of probiotic, antibiotic doses, PPI used, and duration of therapy and they often pooled results with different combinations of probiotics which prevented strain-specific assessments.

Successful treatment of an infectious disease involves selection of the appropriate antimicrobials and determination of the optimum combination of formulation, route of administration, frequency and duration of therapy, and whether adjuvants are needed. The objectives of this study were to address whether probiotics alone would provide a sufficiently high cure rate to be used as monotherapy with a PPI and to identify the cure rate or delta if the effect was additive. We chose *Lactobacillus reuteri* as it is acid resistant and has also been reported to have anti-*H. pylori* activity [10]. Prior studies include a pilot study by Saggioro et al. which compared *L. reuteri* and placebo plus omeprazole 20 mg b.i.d. for 30 days with follow-up by the rapid urease test and histology 4 weeks after the end of therapy [11]. The reported cure rate of *L. reuteri* and omeprazole was 60% (9/15) (95% CI = 32–83%) versus 0% (0/15) (95% CI = 0–21%) with placebo. The study was however published only in abstract format, and additional information has been unavailable despite repeatedly attempting to contact the authors. They also provided no specific *L. reuteri* strain information. We believe strain was most likely *L. reuteri* ATCC 55730 (which is the mother strain to DSM 17938) at a dose of 1 × 10^8^ CFU b.i.d., as it was commercially available in Italy as Reuterin®. Another study by Francavilla et al. compared *L. reuteri* ATCC 55730 given once daily (1 × 10^8^ CFU) for 28 days versus placebo without a PPI. Forty *H. pylori* positive subjects were randomized, and the 13C-UBT and stool antigen test were repeated on day 29. All subjects remained *H. pylori* positive (cure rate of 0%; 95% CI = 0–16.8%) [12]. In another study, Francavilla et al. used a combination of related strains of *L. reuteri* (strains DSM 17938 and ATCC PTA6475, each 10^8^ CFU) versus placebo once daily for 96 days in 100 subjects (50 each) [13]. No PPI was given. The cure rates were 6.0% (95% CI = 1.4–18%) and 2% (95% CI = 0.05–10%) with placebo. This study was based on an open-label pilot study by Dore et al. who gave *L. reuteri* DSM 17938 at a dose of 10^8^ CFU b.i.d. for 60 days plus pantoprazole 20 mg to *H. pylori*-infected adults [14]. No placebo was used. Cure was confirmed by a negative 13C-UBT, 4 or more weeks after the end of therapy with negative tests being reconfirmed after 2 to 3 months. The cure rate was 13.5% (95% CI = 3–35%) for ITT analysis and 14.2% (3/21; 95% CI 3.0–36%) for PP analysis.

## 2. Methods

### 2.1. Study Goal

The goal was to confirm that *L. reuteri* plus a PPI could cure *H. pylori* infections. The primary goal was to test whether increasing the total probiotic dose by decreasing the dosing interval plus a PPI could achieve a clinically acceptable cure rate (e.g., >50%). Success or failure with a high dose, frequent administration, and a long duration would resolve the question about the clinical utility of *L. reuteri* as a standard *H. pylori* therapy as well as provide data about the expected cure rate [15].

### 2.2. Study Design

This study was a 4-week, 2-site, double-blind randomized, parallel-group, placebo-controlled study. Patients scheduled for upper endoscopy for any reason and found to be positive for *H. pylori* infection were invited to enter.

### 2.3. Setting

The study was conducted at the Department of Internal Medicine, University of Sassari, and Department of Interdisciplinary Medicine, University Hospital of Bari, Italy.

### 2.4. Eligibility

Patients older than 18 years scheduled for upper endoscopy for any reason with biopsy specimens and 13C-UBT confirmation of active *H. pylori* infection were invited to participate. Exclusion criteria were severe gastritis (mucosa intensively hyperemic, granular and fragile, and easily bleeding upon the scope touch), peptic ulcer, pregnancy or lactation, malignancy, other clinically significant conditions, alcohol and/or drug abuse, history of allergy to pantoprazole or *L. reuteri*, or taking bismuth compounds, antisecretory drugs, antibiotics, or probiotics during 4 weeks preceding endoscopy.

Written informed consent was obtained from all participants, and the study was approved by the Local Ethics Committees. The protocol was registered at ClinicalTrials.gov: identifier NCT03404440.

### 2.5. Definition of *H. pylori* Infection

Two biopsy specimens were taken from the antrum, one from the angulus and two from the gastric corpus for histology. *H. pylori* infection was defined as the presence of typical histology and bacteria on histologic examination of the gastric biopsies stained by H&E and GIEMSA and a positive 13C-UBT. Posttreatment success was defined by a negative 13C-UBT done 30 to 40 days after completing therapy. The 13C-UBT was performed according to a standardized protocol, the sensitivity and specificity of which have been reported to be of 95%. Breath tests were analysed using a gas isotope ratio mass spectrometer (ABCA, Europe Scientific, Crewe, UK). The test was scored as positive when the Δ-13C-UBT DOB was >5‰. Those with negative 13C-UBT tests were retested after 2 months.

### 2.6. Intervention

In order to achieve the best effect from the *L. reuteri* treatment, based on previous experiences, the dose of probiotic was chosen to be the highest dosage for the longest duration compatible with compliance. The intervention consisted of *L. reuteri* capsules containing *L. reuteri* DSM 17938 plus 2 × 10^8^ CFU *L. reuteri* ATCC PTA 6475 for 2 × 10^8^ CFU (i.e., Gastrus®, BioGaia, Stockholm, Sweden) or placebo seven times daily (total dose 28 × 10^8^ CFU) starting with breakfast and every 2-3 hours independently from the meals along with pantoprazole 20 mg b.i.d. (with meals) all for 28 days. Placebo capsules were identical in shape, colour, and taste to Gastrus capsules but without *L. reuteri*. The placebo was manufactured by BioGaia AB, Stockholm, Sweden, and packaged in identical boxes each containing 30 capsules of placebo or probiotic.

### 2.7. Randomisation and Labelling

Randomisation was computerised using the tool provided by http://randomizer.org which was not otherwise involved in the study labelled active and placebo study products. Randomisation lists were kept in sealed envelopes at the study sites, and a sealed copy was also kept at BioGaia. Subjects were randomized using sealed envelopes with 1 : 1 ratio. Patients were entered into the study sequentially by allocating the lowest available remaining randomisation number to the patient. The code was not broken until all study data had been entered into the database and database locked.

### 2.8. Patient Adherence

Patients were evaluated by a gastroenterologist for adherence and side effects after completing treatment and at follow-up by direct questioning. The study product containers were collected at the day 28 visit, and remaining capsules were counted in order to check adherence. Adherence was considered acceptable in the participant who consumed at least 75% of the allotted doses. Patients that were nonadherent but had consumed at least one full day of doses (i.e., 7 capsules + 2 PPI) were included in the ITT analysis.

In order to assess side effects, participants were asked to complete the short-form Leeds dyspepsia (SF-LDQ) questionnaire after inclusion but before taking the first dose of study product, at 4 weeks (end of treatment) and at 8-9 weeks (at follow-up 13C-UBT) [16, 17]. The questionnaire included symptoms such as nausea, regurgitation, indigestion, and heartburn and were graded as mild (did not limit daily activities), moderate (limited daily activities to some extent), or severe (made daily activities all but impossible).

### 2.9. Statistical Analysis

It is accepted that there is no placebo response in *H. pylori* therapy, and thus, the sample size was calculated assuming that the spontaneous eradication of *H. pylori* was <5%. To be considered as an effective stand-alone therapy, the eradication rate on active treatment was required to exceed 50%. At least 23 patients in each group were considered sufficient for a power of 90% and a confidence level of 95%. To account for dropouts, the sample size of 30 per group was chosen. The primary efficacy parameter was the number of patients with *H. pylori* infection eradicated at 9 weeks assessed by Δ-13C-UBT <5‰. Changes in SF-LDQ at 4 and 9 weeks were compared to baseline and between groups. Analysis of *H. pylori* eradication efficacy was performed on an ITT, basis including all eligible subjects enrolled in the study, and on PP, basis excluding subjects lost to follow-up and protocol violations. No placebo is required with *H. pylori* because spontaneous cures either do not occur or are extremely rare within the time frame; a placebo was used in that there were no data available that the probiotic therapy alone would be effective such that a truly informed consent could be obtained [15, 18]. We informed the subjects of the chance to be assigned to the placebo group meaning that the placebo regimen had no chance of curing *H. pylori*. However, they would remain under care until successful *H. pylori* eradication.

## 3. Results

### 3.1. Subjects

Fifty-six subjects were entered and received at least one dose of study drug; 48 completed the treatment including 34 women and 14 men, and mean age = 49 years (Table 1). Fifty-three were naïve, 2 were previously treated in the active group, and 1 was treated in the placebo group. Recruitment was halted after 56 subjects because the overall low cure rate ensured that the minimum goal of 50% could not be achieved if all possible subjects were to be entered. Of the 56 subjects, 28 were randomly assigned to the active treatment group and 28 to the placebo. Baseline features of subjects were similar (Table 1).

### 3.2. Subject Adherence

**Figure 2:**
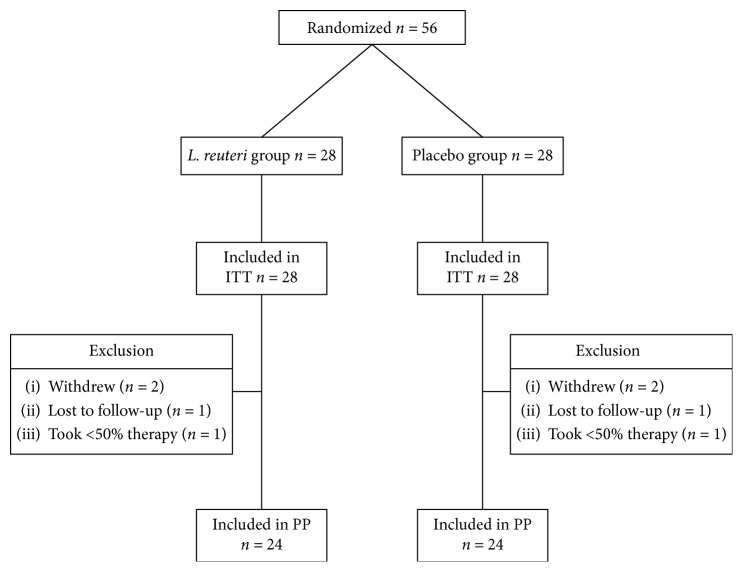
Disposition of study patients. PP = per protocol; ITT = intention to treat.

Figure 2 is the flow diagram showing the outcome of each subject entered. The overall tolerability was good. Among 28 subjects, 4 withdrew because of intercurrent illnesses and 2 were lost to follow-up. One in each group who completed the study took less than 50% of caps. Excellent adherence defined as >75% (100%) was achieved in 24 of 25 subjects (96%) completing therapy in each of the active and placebo groups.

### 3.3. Side Effects

**Figure 3:**
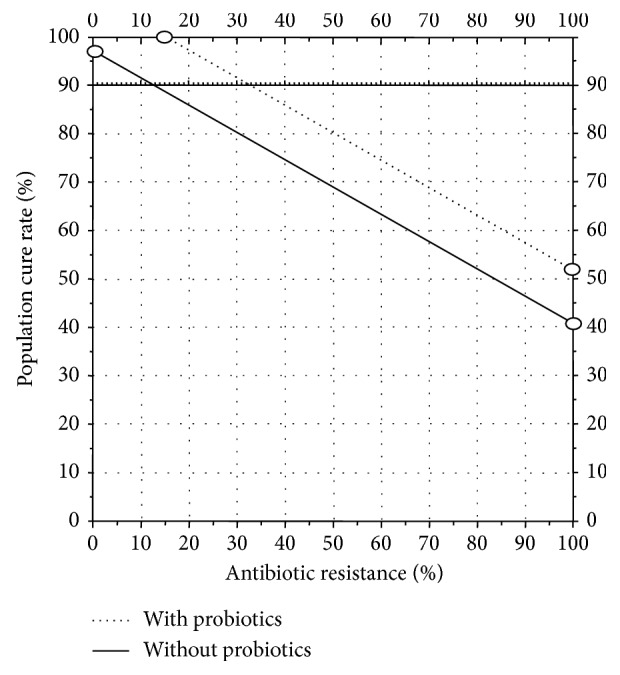
Hp-treatment nomogram showing theoretical cure rates with 14-day triple therapy with standard dose PPI (e.g., 40 mg of omeprazole b.i.d.) in the absence and presence of resistance. This would increase the population cure rate in the “all clarithromycin resistant” subgroup (PPI + amoxicillin) from 20 to 40%. Based on an increase in the cure rate of 12% with probiotic, the addition of probiotic adjuvant therapy would increase the point where the cure rates fell below 90% from approximately 13% (solid line) to 33% (dotted line).

**Figure 4:**
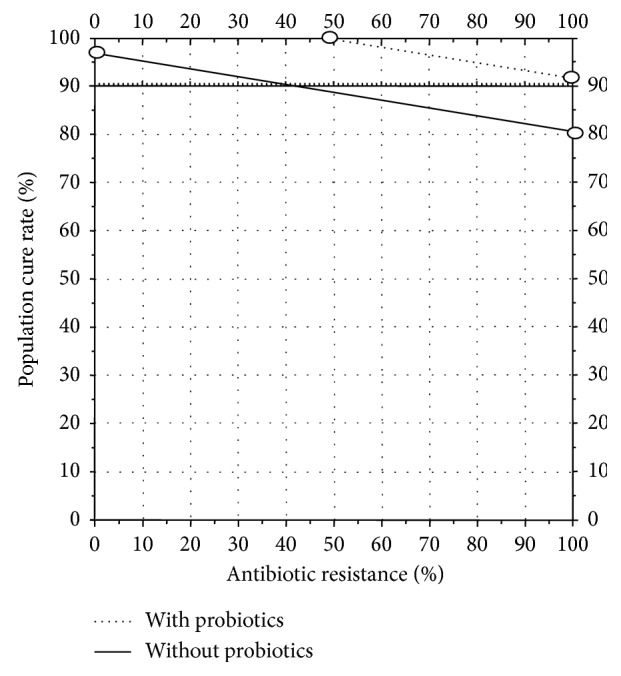
Hp nomogram showing theoretical cure rates with 7 day vonoprazan 20 mg b.i.d. plus amoxicillin 1 g b.i.d. in the absence and presence of resistance [19]. Based on an increase in the cure rate of 12% with probiotic, the addition of probiotic adjuvant therapy would increase the point where the cure rates fell below 90% from approximately 42% (solid line) to 100% (dotted line) such that dual vonoprazan-amoxicillin therapy would be sufficient and clarithromycin would become unnecessary.

Side effects were recorded, and the SF-LDQ questionnaire was administered. Results were available for all subjects who completed the study (Figures 3 and 4). The questionnaire was completed at the time of the enrolment and at the final UBT. The most common side effect was diarrhea and was more prevalent in the active group (3/24 versus 1/24; *P*=0.296).

There was a very modest improvement about symptoms between the first and second visit and no difference between the first and third visits. The mean of symptoms frequency and interference sum for subjects in the active versus placebo group was at the first visit: 19.3 versus 19.8; at the second visit: 15.3 versus 17.9; and at the third visit: 15.2 versus 18.8, respectively.

### 3.4. Eradication of *H. pylori* Infection

Among 56 subjects enrolled, there were four dropouts (Figure 2). The causes of dropout included need of antibiotic treatment for pneumonia (1), urinary tract infection (1), dental abscess (1), and surgery (2). Two additional subjects were lost to the follow-up: one for nausea and vomiting because of labyrinthitis (Table 2). The cure rate in the active group was 12.5% (3/24, 95% CI 2.6–32.3%) for PP analysis and for ITT analysis was 10.7% (3/28, 95% CI 2.2–28.2%). Unexpectedly, one placebo patient was cured as reflected by a negative UBT which was repeated after 8 weeks. The resulting cure rate in the placebo group for PP analysis was 4.1% (1/24, 95% CI 0.1–21.1%), and for ITT analysis 3.5% (1/28, 95% CI 0.0–18.3%).

## 4. Discussion

Recent meta-analyses have compiled the results of the many studies comparing the data obtained by adding a probiotic or combination of probiotics or placebo to *H. pylori* antimicrobial therapy [3–8]. As noted above, overall results involving more than 4,000 subjects have shown a mean and significant increase in the ITT eradication rate of 12.2% (95% CI 9.1–15.3%) compared to placebo [4]. As noted earlier, the studies typically involved populations in which antimicrobial resistance had compromised the effectiveness of traditional triple therapies, thus reducing the cure rates and allowing any improvement in eradication to be visualized. The mean increase in the cure rate was from 68.2% to 78.5% in 30 randomized clinical trials involving more than 4,000 patients (4,302 per protocol and 4,415 intention to treat) [4]. The mean increase per protocol was 12.2% (95% CI = 9–15%) for ITT and 14.1 (95% CI = 6–17%) for PP [4]. A variety of strains of probiotics have been reported to be effective including *Lactobacillus*, *Bifidobacterium*, *Saccharomyces*, and mixtures including *Lactobacillus acidophilus*/*Bifidobacterium animalis* among others [4–6]. There is no evidence of a dose-response effect or duration effect for therapies lasting 7 days or over.

Success has also been reported in both Europe and Asia without regional differences and with different *H. pylori* eradication therapies including 7-day and 14-day triple therapy as well as one PPI, amoxicillin, clarithromycin, amoxicillin, and bismuth containing regimen [5]. The overall data suggests that the increase in success is independent of the cure rate of the individual study (i.e., the effect of probiotics is additive). The current study and the prior study by Dore et al. [14] achieved similar results. Overall, this study confirmed that *L. reuteri* plus a PPI is unable to achieve a sufficiently high cure rate to be used as a stand alone anti-*H. pylori* regimen. However, the results are consistent with the hypothesis that a probiotic adjuvant can be expected to increase the overall cure rate by approximately 10–14%. Whether this improvement is considered clinically relevant based on the overall cure rate achieved with the other components of therapy is dependent on the cure rates of the susceptible and resistance populations and the prevalence of resistance. A clinically meaningful improvement requires that the ∼12% additive effect of probiotics should achieve a final cure rate of 90% or greater. This would require that the overall cure rate must be at least ∼80% without the addition of the probiotic.

For example, Figure 3 shows the effect of additive effect of a probiotic that adds 12% to the cure rate. In this example, the cure rate with clarithromycin-resistant strains with a 14-day triple therapy using a high dose of PPI such as 40 mg of rabeprazole (72 mg omeprazole equivalent) b.i.d. [20]. The cure rate would fall below 90% when the proportion with resistance exceeded 13%. With the addition of a probiotic, the cure rate would remain at 90% or greater until the proportion with resistance increased to approximately 33%. Increasing the cure rate with resistant infections such as with vonoprazan or multiple high-dose PPI therapy might allow the cure rate to remain above 90% irrespective of the proportion with clarithromycin resistance and to possibly even eliminate clarithromycin altogether [19].

### 4.1. What Remains to Be Done

As with any therapy, doses, formulation, frequency of administration, administration in relation to meals, and use of adjuvants such as PPIs may all be important. As described above, prior studies with *L. reuteri* given without antibiotics suggest that low dose (e.g., 1 × 10^8^ CFU) given once a day without a PPI was less effective [12] than twice a day therapy which cured 6.0% (95% CI = 1.4–18%) [13]. In contrast, twice a day plus low-dose PPI (9 mg omeprazole equivalent) cured 13.5% (95% CI = 3–35) [14, 20]. However, PPIs are typically a component of *H. pylori* eradication therapy. It is unclear whether the probiotic effect is enhanced by increased antisecretory activity. As noted above, addition of a probiotic to triple therapy produced similar improvement with therapies of 7 to 14 days [5], and 14-day duration has been recommended as the preferred treatment duration by all recent *H. pylori* consensus conferences [21–23]. Another reason to use a 14-day duration is because the effectiveness of PPI-amoxicillin dual therapy is markedly influenced by duration and antisecretory effect (reviewed in [24]) and by the potency of the antisecretory drug [20, 25]. The effect of the coadministration of bismuth is as yet unclear.

Because the overall improvement with probiotics is small, it can only be measured accurately in comparative studies if the two groups are highly similar in terms of the proportion with susceptible and resistant strains. To ensure comparable starting points, future studies examining whether probiotics improve overall cure rates in comparative trials will also need to assess cure rates for both the susceptible and resistant populations to ensure that the two groups are comparable in that differences in the study groups can greatly influence the apparent effect of the addition of probiotics. Without such data, one is required to use pooled data and meta-analyses to assess the effects, but that approach was unable to determine whether it was additive or synergistic or what factors affected it.

## 5. Conclusion

Our findings show that *L. reuteri* plus a PPI was unable to provide a clinically important rate of *H. pylori* eradication. However, we confirmed data from meta-analyses of improvement in cure rates with triple therapy in the presence of *H. pylori* resistance. We confirmed that the overall improvement in the cure rate is low (e.g., 12.5%) and were able to show that the effect was independent of the use of antibiotics or the presence of resistant and is most consistent with the effect being additive (i.e., approximately 12% increase of the cure rate without the probiotic).

The overall data suggest that at least 2 capsules of Gastrus® would likely be required. The optimum duration is still unknown, but if the data from the clinical trials are a guide, a duration of 14 days or possibly less should suffice. There appears to be no advantage of higher dose or more frequent administration. Future studies will be required to identify the minimal and most cost-effective regimen.

## Figures and Tables

**Table 1:** Baseline characteristics of the two groups.

Characteristic	Active group^a^	Placebo group^b^
No. of subjects	28	28
Male/female	9/19	11/17
Mean age (year)	51.2	54.7
Mean body mass index	23.2	24.5
Smokers	7	3
Ex-smokers	2	4
NSAID users	3	3
Naïve for *H. pylori* treatment	27	26

^a^Subjects treated with Gastrus® capsules (2 × 10^8^ CFU *L. reuteri* DSM 17938 + 2 × 10^8^ CFU *L. reuteri* ATCC PTA 6475) with pantoprazole 20 mg, b.i.d. ^b^Subjects treated with placebo with pantoprazole 20 mg, b.i.d.

**Table 2:** Intervention status of subjects enrolled in the trial.

Treatment	Active	Placebo
Received intervention as allocated	28	28
Withdrew from study	2	2
Lost to follow-up	1	1
Less than 50% of intervention	1	1
Completed trial	24	24
Treatment failures	21	23
Cure rate ITT	3/28 (11%)	1/28 (3.6%)
95% CI	2.2–28.2	0.0–18.34
Cure rate PP	2/24 (12.5%)	1/24 (4%)
95% CI	2.6–32.3	0.1–21.1

ITT: intention-to-treat analysis; CI: confidence interval; PP: per protocol analysis.

## Data Availability

Data are available upon request.
